# Genome-Wide Analysis of Experimentally Evolved Candida auris Reveals Multiple Novel Mechanisms of Multidrug Resistance

**DOI:** 10.1128/mBio.03333-20

**Published:** 2021-04-05

**Authors:** Hans Carolus, Siebe Pierson, José F. Muñoz, Ana Subotić, Rita B. Cruz, Christina A. Cuomo, Patrick Van Dijck

**Affiliations:** a VIB Center for Microbiology, Leuven, Belgium; b Department of Biology, KU Leuven, Leuven, Belgium; c Broad Institute of MIT and Harvard, Cambridge, Massachusetts, USA; Vallabhbhai Patel Chest Institute

**Keywords:** *Candida auris*, multidrug resistance, microevolution, whole-genome sequencing, amphotericin B, antifungal agents, caspofungin, drug resistance evolution, experimental evolution, fluconazole, genome analysis

## Abstract

Candida auris is globally recognized as an opportunistic fungal pathogen of high concern, due to its extensive multidrug resistance (MDR). Still, molecular mechanisms of MDR are largely unexplored. This is the first account of genome-wide evolution of MDR in C. auris obtained through serial *in vitro* exposure to azoles, polyenes, and echinocandins. We show the stepwise accumulation of copy number variations and novel mutations in genes both known and unknown in antifungal drug resistance. Echinocandin resistance was accompanied by a codon deletion in *FKS1* hot spot 1 and a substitution in *FKS1* “novel” hot spot 3. Mutations in *ERG3* and *CIS2* further increased the echinocandin MIC. Decreased azole susceptibility was linked to a mutation in transcription factor *TAC1b* and overexpression of the drug efflux pump Cdr1, a segmental duplication of chromosome 1 containing *ERG11*, and a whole chromosome 5 duplication, which contains *TAC1b*. The latter was associated with increased expression of *ERG11*, *TAC1b*, and *CDR2* but not *CDR1*. The simultaneous emergence of nonsense mutations in *ERG3* and *ERG11* was shown to decrease amphotericin B susceptibility, accompanied with fluconazole cross-resistance. A mutation in *MEC3*, a gene mainly known for its role in DNA damage homeostasis, further increased the polyene MIC. Overall, this study shows the alarming potential for and diversity of MDR development in C. auris, even in a clade until now not associated with MDR (clade II), stressing its clinical importance and the urge for future research.

## Abstract (precis)

Candida auris is a recently discovered human fungal pathogen and has shown an alarming potential for developing multi- and pan-resistance toward all classes of antifungals most commonly used in the clinic. Currently, C. auris has been globally recognized as a nosocomial pathogen of high concern due to this evolutionary potential.

## INTRODUCTION

Over the course of a decade since its discovery (1), Candida auris has emerged in at least 39 countries in every inhabited continent (2), occasionally causing health care-associated outbreaks of lethal candidiasis (3). C. auris is substantially different from any other *Candida* species studied so far, as it behaves like a true multidrug-resistant (MDR) nosocomial pathogen (cf. methicillin-resistant Staphylococcus aureus, MRSA) (3). This was illustrated by the U.S. Centers for Disease Control and Prevention (CDC) in 2019 when they listed C. auris as the first fungus among urgent antimicrobial resistance threats (4). C. auris can become resistant to each drug and each combination of drugs from the three major antifungal drug classes, the azoles (e.g., fluconazole), echinocandins (e.g., caspofungin), and polyenes (e.g., amphotericin B).

Various clinical isolate screening reports indicate fluconazole resistance in over 80% (5–9) and amphotericin B resistance in up to 30% of the isolates tested (6, 7). Echinocandin resistance is less common, found in 2 to 10% in some screenings (6–8, 10), but it is alarmingly on the rise (11, 12). Overall, about 90% of the C. auris isolates are estimated to have acquired resistance to at least one drug, while 30 to 41% are resistant to two drugs, and roughly 4% are pan-resistant (resistance to the three major antifungal drug classes) (4, 7). These numbers show an unprecedented potential to acquire MDR, unlike any other pathogenic *Candida* species (3, 12, 13). The molecular mechanisms of antifungal drug resistance in C. auris, especially for amphotericin B resistance and MDR, are still poorly understood. Hundreds of resistant C. auris strains have been sequenced, and their decreased drug susceptibility for azoles and echinocandins has been associated with a few mutations in genes known to be involved in drug resistance. Still, the high levels of resistance and extensive MDR in some strains cannot be explained through the limited number of resistance-conferring mutations described so far (3, 7).

Azole resistance has been linked to three single nucleotide polymorphisms (SNPs) (7–9, 14) and an increased copy number (9, 15) of *ERG11*, the gene encoding the fluconazole target lanosterol 14-α-demethylase. The ATP binding cassette (ABC) transporter Cdr1 was proven to act as an efflux pump of azoles in C. auris (16–18), and another study suggests that gain of function (GOF) mutations in *TAC1b* can underly this mode of action (16). A recent study suggests that azole resistance can be the result of a duplication of chromosome V, which contains several genes involved in drug resistance and ergosterol biosynthesis (19). Reduced echinocandin susceptibility in C. auris was previously only linked to SNPs substituting amino acids S639 (9, 12, 20) and F635 (21) and a deletion of F635 (22) in Fks1, which is the echinocandin target, β(1,3) d-glucan synthase. The polyene amphotericin B works by sequestering ergosterol and induction of oxidative stress (23), rather than inhibiting a specific enzyme, and therefore, amphotericin B resistance is among the least understood drug resistance mechanisms in C. auris and *Candida* spp. in general (12, 23). So far, only an increased expression of genes involved in ergosterol biosynthesis (i.e., *ERG1*, *ERG2*, *ERG6*, and *ERG16*) (15) and SNPs in *ERG2* (24), *FLO8*, and an unnamed membrane transporter-encoding gene (25) have been linked to amphotericin B resistance in C. auris (12, 20).

Overall, few studies have actually been able to validate the proposed drug resistance mechanisms in C. auris (16, 18, 26, 27), presumably because of the lack of an optimized gene-editing system. Here, we apply a strategy of serial transfer-based experimental evolution with the ability to trace back the emergence—and validate the cumulative effect—of single mutations or copy number changes throughout the evolutionary process. By designing allele-specific PCR primers, the presence or absence of specific mutations could easily be screened for by PCR on multiple single clones in the daily evolving populations. Doing so, we tracked down the emergence of 10 nonsynonymous mutations in 8 genes, evolved in 5 separate evolutionary lineages. In this study, we investigated MDR evolution in a clade II C. auris strain, which is understudied compared to other clades (28) and has been suggested to be less prone to drug resistance development (28, 29). Previously, five different clades (clades I to V, i.e., the South Asian, East Asian, African, South American, and Iranian clades, respectively) of C. auris were identified, each phylogenetically separated by thousands of SNPs (7, 30) and often associated with clade-specific virulence and/or drug resistance tendencies (9, 28, 29). This study shows that clade II C. auris can rapidly acquire MDR *in vitro*, and its mechanisms of resistance provide fundamental new insights on how resistance can be acquired by C. auris. Finally, our study presents both the power and challenges of using *in vitro* experimental evolution to discover molecular mechanisms of (multi)drug resistance.

## RESULTS

### C. auris clade II can acquire multidrug resistance rapidly *in vitro*.

**FIG 1:**
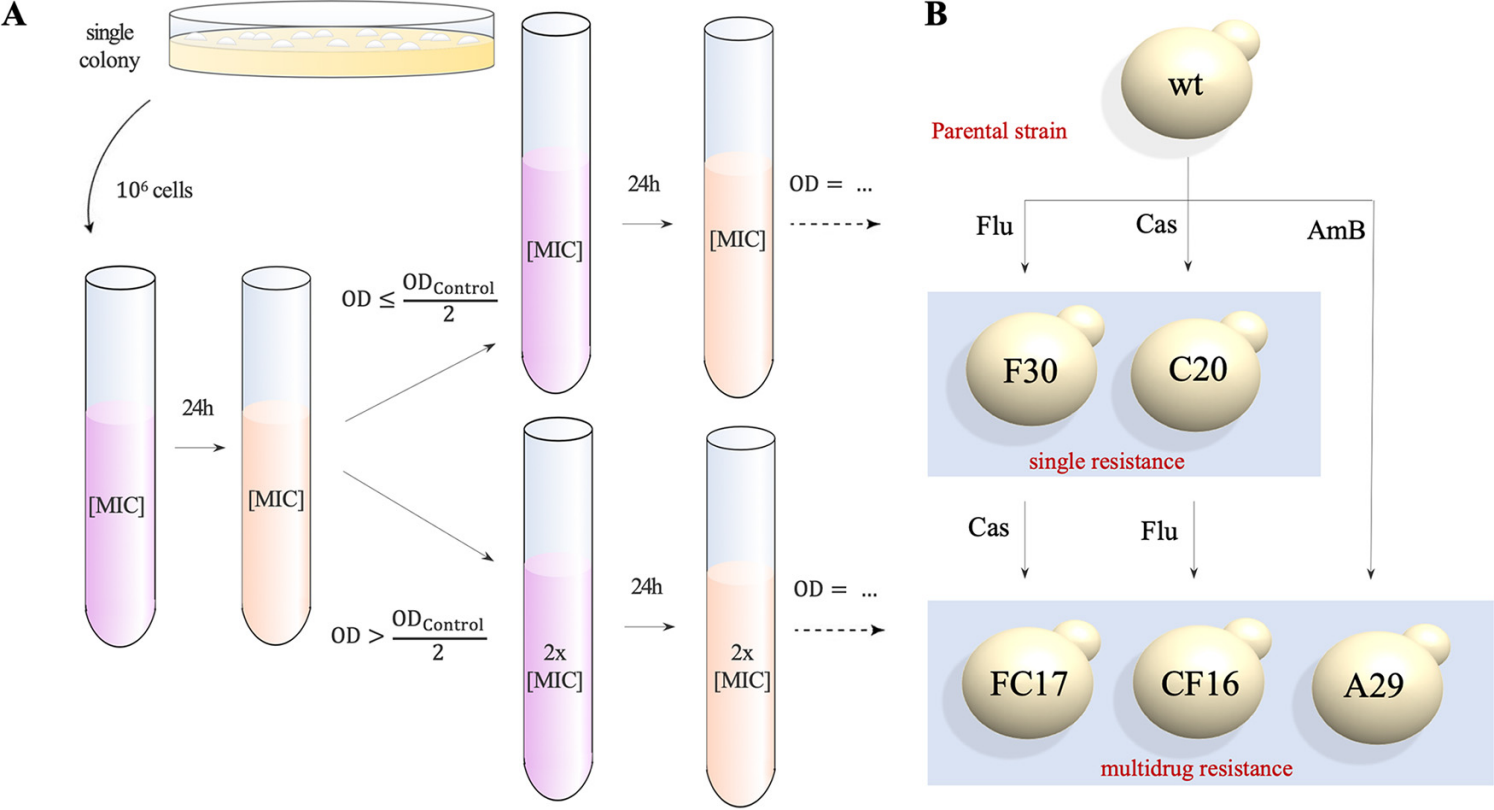
Schematic overview of the *in vitro* experimental evolution assay. (A) A single colony is cultured in RPMI-MOPS medium (2% glucose) for 24 h at 37°C after which a standardized inoculum (10^6^ cells) is resuspended in medium containing no drug (control), the drug at a concentration of 2× MIC_50_, 1× MIC_50_, and 0.5× MIC_50_ (shown here) of the particular starting strain. Daily, the culture is rediluted (1/10) in fresh RPMI-MOPS medium (2% glucose) with a concentration of drug based on the OD_600_ of the control culture (see Materials and Methods). All strains were evolved in triplicate. Daily aliquots of evolving populations were stored in RPMI-MOPS medium containing 25% glycerol at −80°C for later analysis. (B) Ancestry of the five evolved strains that were sequenced. WGS was performed on a single colony. The name of each strain represents the experimental treatment (letter) and day of isolation (number), respectively.

A single colony of C. auris strain B11220, the original type strain described by Satoh et al. (1) from Japan in 2009, was subjected to an *in vitro* experimental microevolution assay as depicted and described in Fig. 1A and Materials and Methods, respectively. This parental progenitor (further referred to as the wild type [wt]) proved to be pan-susceptible (determined by MIC or MIC_50_, see Materials and Methods) to fluconazole (MIC_50_, 1 μg/ml), caspofungin (MIC_50_, 0.125 μg/ml), and amphotericin B (MIC_50_, 0.5 μg/ml). Based on these MIC_50_ values, the wild type strain was exposed (in triplicate) to three concentrations of each drug as follows: 2× MIC_50_, 1× MIC_50_, and 0.5× MIC_50_, or no drug, representing three selective pressures and a control, respectively. Serial transfer with conditional drug treatment (Fig. 1A) was maintained for 30 days or until drug resistance became evident from regular MIC testing. An overview of the ancestry of the evolved strains is depicted in Fig. 1B.

**FIG 2:**
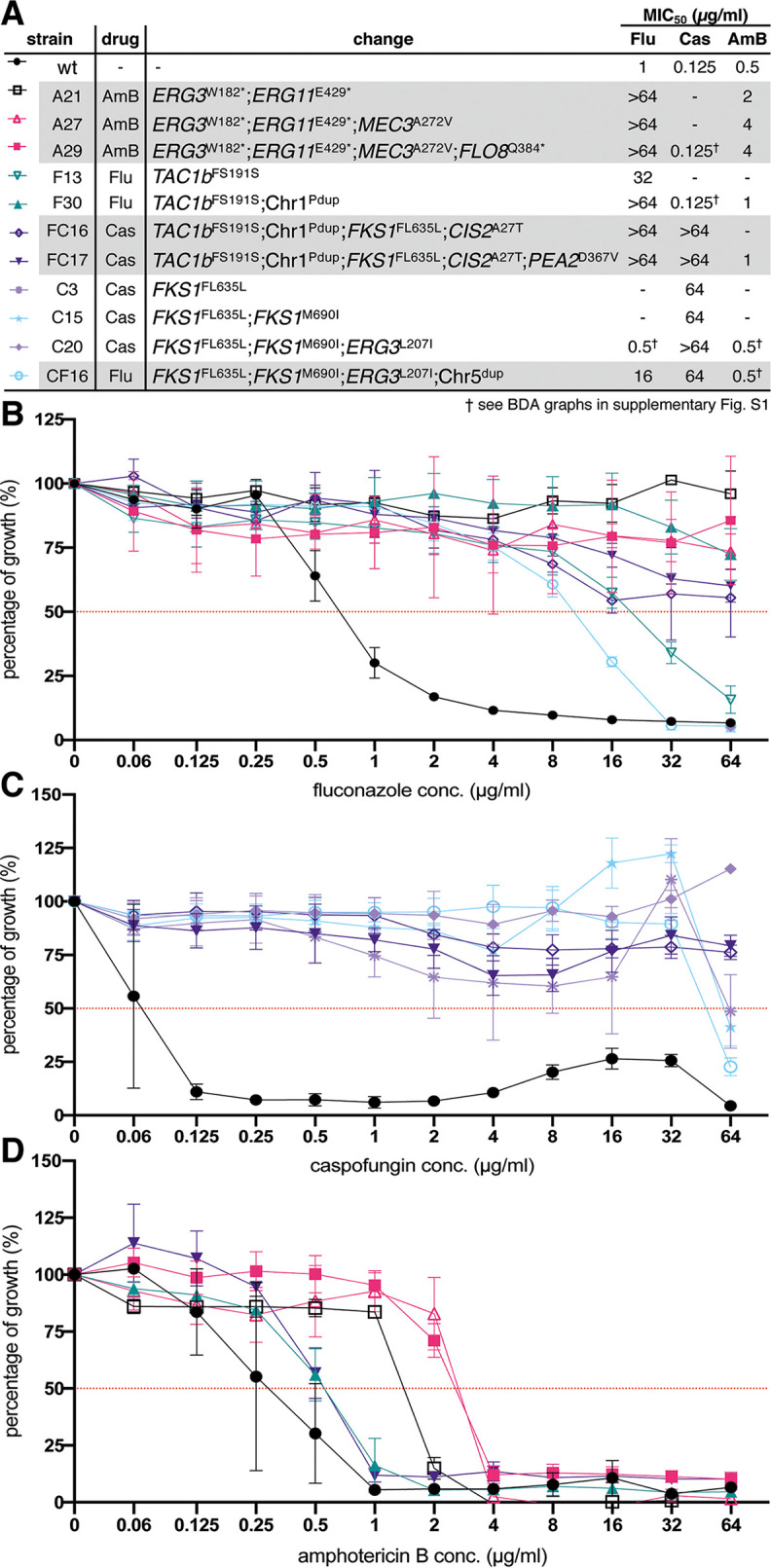
Resistance profiles of endpoint and intermediate evolved strains. (A) Summary of MIC_50_ values and associated mutations/CNVs for each endpoint strain and divergent intermediate strain. (B to D) Growth profiles of evolved strains relative to the wild-type strain (wt) in a broth dilution assay (BDA) of fluconazole (B), caspofungin (C), and amphotericin B (D). The percentage of growth was calculated from growth without drug and based on OD_600_ measurements after 48 h of incubation at 37°C. Each data point and its standard deviation is calculated from 3 biological repeats, each represented by the mean of 2 technical repeats. Pdup: partial duplication, dup: duplication. Resistance profiles of endpoint evolved strains for all drugs are found in supplemental material Fig. S1.

Five strains were evolved and sequenced as follows: F30, C20, A29, FC17, and CF16. Strain B11220 (wild type) was exposed to fluconazole (F-lineage), caspofungin (C**-**lineage), and amphotericin B (A-lineage). Next, these single resistant strains were exposed to a second drug to acquire multidrug resistance, yielding the FC-lineage for the F (fluconazole-resistant) strain that was given caspofungin, and the CF-lineages for the C (caspofungin-resistant) strain that was given fluconazole, respectively. The name of each strain represents the experimental lineage (letter which refers to the treatment) and day of isolation (number), respectively. Figure 2 and Fig. S1 (supplemental material) show the MIC_50_ values for each drug of each endpoint evolved strain (F30, C20, A29, FC17, and CF16). The length of the evolution experiment ranged from 16 (CF-lineage) to 30 days (F-lineage), although later it was shown that resistant clones emerged quite early (e.g., after 3 days in C-lineage; see Fig. 2).

### Allele-specific PCR is an effective method for tracing back the emergence of mutations during evolution in serial isolates.

**FIG 3:**
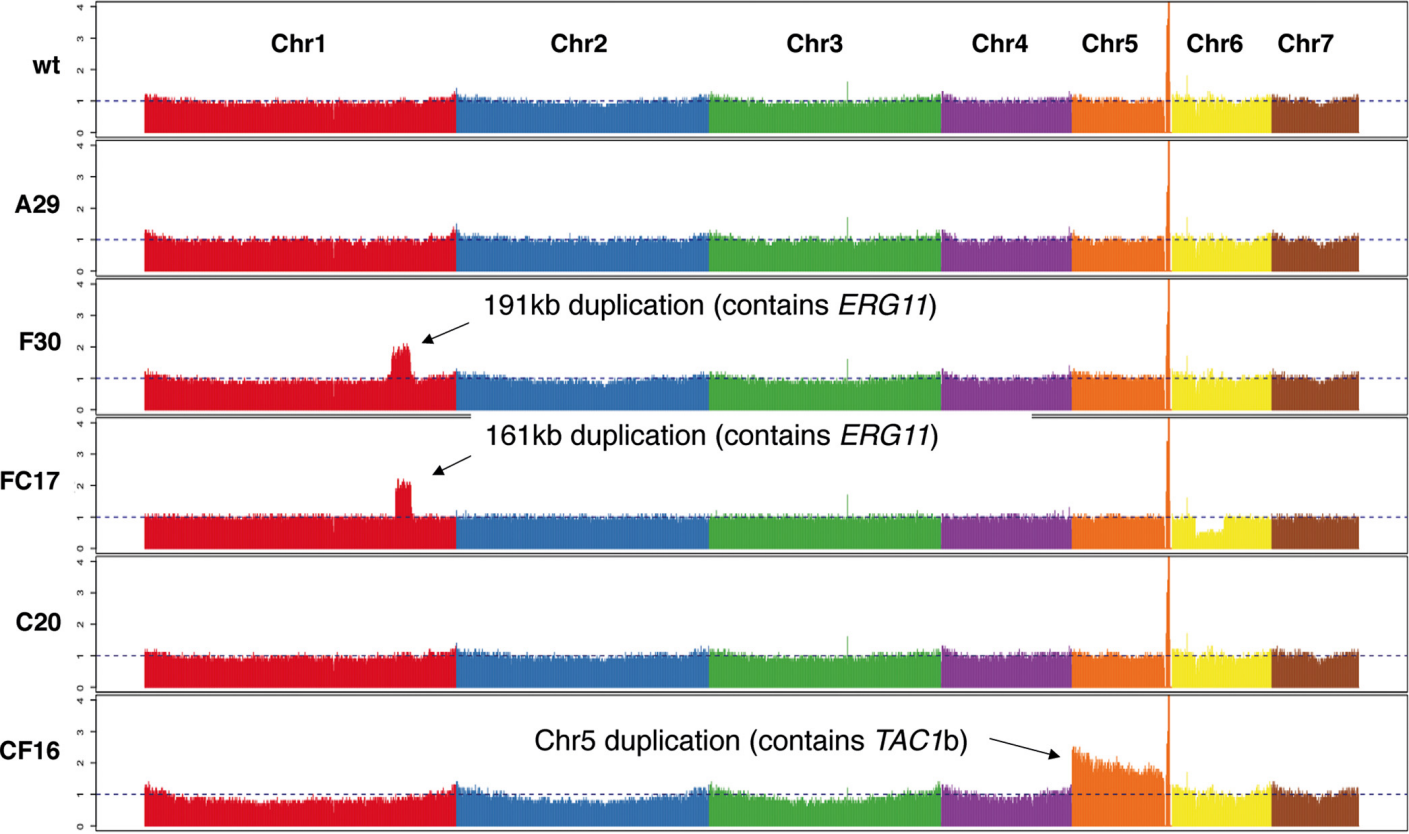
Coverage plot of whole-genome sequencing of endpoint evolved strains. The coverage displayed is calculated by normalizing the average coverage depth per 5-kb window. Each color represents 1 chromosome (from left to right, chromosomes 1 to 7). Indicated are the significant duplication in chromosome 1 (Chr1) in strain F30 and FC17 and chromosome 5 (Chr5) in strain CF16.

After microevolution, whole-genome sequencing of the wild type, F30, C20, A29, FC17, and CF16 strains showed the acquisition of 10 nonsynonymous mutations (listed in Table 1) and two different aneuploidies (shown in Fig. 3 and Fig. S3). All mutations except the deletion of F635 in *FKS1* (see below) were novel to C. auris based on literature review and comparison with a set of 304 globally distributed C. auris isolate sequences representing clades I, II, III, and IV (9). The impact and/or cumulative effect of individual mutations and copy number variations (CNVs) will be discussed in the following paragraphs. To validate the effect of single mutations or aneuploidies in strains that harbored more than one mutation, we applied a screening strategy on evolving serial isolates using allele-specific PCR (AS-PCR). AS-PCR primers were designed as described by Liu et al. (31), implementing a specific mismatch at the third position of the 3′ end of the allele-specific primer to increase specificity. An overview of the universal and allele-specific primers used to perform AS-PCR, is given in Table S1. The specificity and sensitivity of all AS-PCR primers was assessed by performing temperature gradient PCRs on serial dilutions of the reference DNA template (for one example, see Fig. S2). Cells were recultured from the −80°C collection of daily stored aliquots (serial isolates or “populations”), and AS-PCR was performed on genomic DNA (gDNA) extracted from a maximum of 30 single clones of each population. After confirmation of the emergence of a mutation of interest, alleles were verified by sequencing a ±1,000-bp region spanning the allele of interest. Primers used for PCR and sequencing are given in Table S1. Next, the effect of single mutations on the drug susceptibility was analyzed by comparing MIC values of the serial isolates through broth dilution assays (BDA; see “Antifungal Susceptibility Testing” in Materials and Methods). Figure 2 shows the impact of each individual mutation on the MIC for the drug of interest for each lineage evolved, except for the A-lineage (i.e., amphotericin B resistance evolution), in which the mutations in *ERG3* and *ERG11* (Table 1) were either present or absent simultaneously in all colonies that were checked.

**TABLE 1:** All nonsynonymous mutations identified in the endpoint evolved strains^*a*^

Strain	Change	Type of change	Gene ID (B11220)	Gene ID (B8441)	Ortholog
A29	tgG/tgA|W182*	Nonsense	CJI96_002270	B9J08_003737	*ERG3*
	Gag/Tag|E429*	Nonsense	CJI96_001197	B9J08_001448	*ERG11*
	Cag/Tag|Q384*	Nonsense	CJI96_001121	B9J08_000401	*FLO8*
	gCg/gTg|A272V	Missense	CJI96_001637	B9J08_003102	*MEC3*
F30	ttcagt/agt|FS191S	Codon deletion	CJI96_004335	B9J08_004820	*TAC1b*
FC17	ttcagt/agt|FS191S	Codon deletion	CJI96_004335	B9J08_004820	*TAC1b*
	ttcttg/ttg|FL635L	Codon deletion	CJI96_001351	B9J08_000964	*FKS1*
	gAt/gTt|D367V	Missense	CJI96_001286	B9J08_001356	*PEA2*
	Gca/Aca|A27T	Missense	CJI96_001769	B9J08_003232	*CIS2*
C20	atG/atA|M690I	Missense	CJI96_001351	B9J08_000964	*FKS1*
	ttcttg/ttg|FL635L	Codon deletion	CJI96_001351	B9J08_000964	*FKS1*
	Cta/Ata|L207I	Missense	CJI96_002270	B9J08_003737	*ERG3*
CF16	atG/atA|M690I	Missense	CJI96_001351	B9J08_000964	*FKS1*
	ttcttg/ttg|FL635L	Codon deletion	CJI96_001351	B9J08_000964	*FKS1*
	Cta/Ata|L207I	Missense	CJI96_002270	B9J08_003737	*ERG3*

a Nucleotide and amino acid changes compared to the reference parent strain genome (wild type) are displayed. Genes were identified based on orthologues annotated in the C. auris B8441 genome sequence as provided at http://www.candidagenome.org/.

10.1128/mBio.03333-20.1 FIG S1 Resistance profiles of endpoint evolved strains. Growth profiles in a broth dilution assay (BDA) of the endpoint evolved strains (A29, F30, FC17, C20, CF16) and control strain (wt) for fluconazole (A), caspofungin (B), and amphotericin B (C). The percentage of growth was calculated from growth without drug and based on OD_600_ measurements after 48 h of incubation at 37°C. Each data point and its standard deviation is calculated from 3 biological repeats, each represented by the mean of 2 technical repeats. (D) Summary of MIC_50_ values for each strain and indication of (relatively low [↑] or high [↑↑]) increase or decrease (↓) of MIC in respect to the parental strain (wt) MIC. Download FIG S1, TIF file, 0.2 MB. Copyright © 2021 Carolus et al. 2021 Carolus et al. https://creativecommons.org/licenses/by/4.0/ This content is distributed under the terms of the Creative Commons Attribution 4.0 International license.

10.1128/mBio.03333-20.2 FIG S2 Example of the temperature specificity range of allele-specific primers. Here, a temperature gradient PCR was performed using the *MEC3* allele-specific primers on gDNA of the wild-type strain (wt) and strain A29, containing the gCg/gTg|A272V mutation in *MEC3* (see Table 1). Primer pairs “*WT*: and “Δ” indicate the primers targeting the wild-type allele (*CauMEC3_B11220_PCR/Seq1_F* and *CauMEC3_SNP_A272A_R*) and mutated allele (*CauMEC3_B11220_PCR/Seq1_F* and *CauMEC3_SNP_A272V_R*), respectively, and are given in Table S1. The green grid indicates a primer-specific temperature range (63 to 64°C). Download FIG S2, TIF file, 0.1 MB. Copyright © 2021 Carolus et al. 2021 Carolus et al. https://creativecommons.org/licenses/by/4.0/ This content is distributed under the terms of the Creative Commons Attribution 4.0 International license.

10.1128/mBio.03333-20.3 FIG S3 Copy number variation quantification of *TAC1b* (marker on the Chr5 duplication in the CF-lineage; see Fig. 3), *ERG11* (marker on the segmental duplication of Chr1 in F- and FC-lineage; see Fig. 3), and *ACT1* (reference) by qPCR on gDNA. CNVs were determined for 1 biological repeat represented by 3 technical repeats. Download FIG S3, TIF file, 0.03 MB. Copyright © 2021 Carolus et al. 2021 Carolus et al. https://creativecommons.org/licenses/by/4.0/ This content is distributed under the terms of the Creative Commons Attribution 4.0 International license.

10.1128/mBio.03333-20.6 TABLE S1 All primers used in this study. Primers are arranged per marker. “Purpose” indicates whether the primer was used for PCR and sequencing (PCR/seq), CNV or expression analysis (qPCR), or allele-specific PCR (AS). Primer pairs for AS-PCR consist of a universal PCR-sequencing primer (indicated by “PCR/seq/AS”) or universal AS-primer (indicated by “AS”) and one allele-specific primer (indicated by “AS-wt” for the wild-type allele and “AS-mt” for the mutant allele). Download Table S1, DOCX file, 0.02 MB. Copyright © 2021 Carolus et al. 2021 Carolus et al. https://creativecommons.org/licenses/by/4.0/ This content is distributed under the terms of the Creative Commons Attribution 4.0 International license.

### High-level echinocandin resistance without fitness discrepancies evolved through mutations in *FKS1* and *ERG3*.

**FIG 4:**
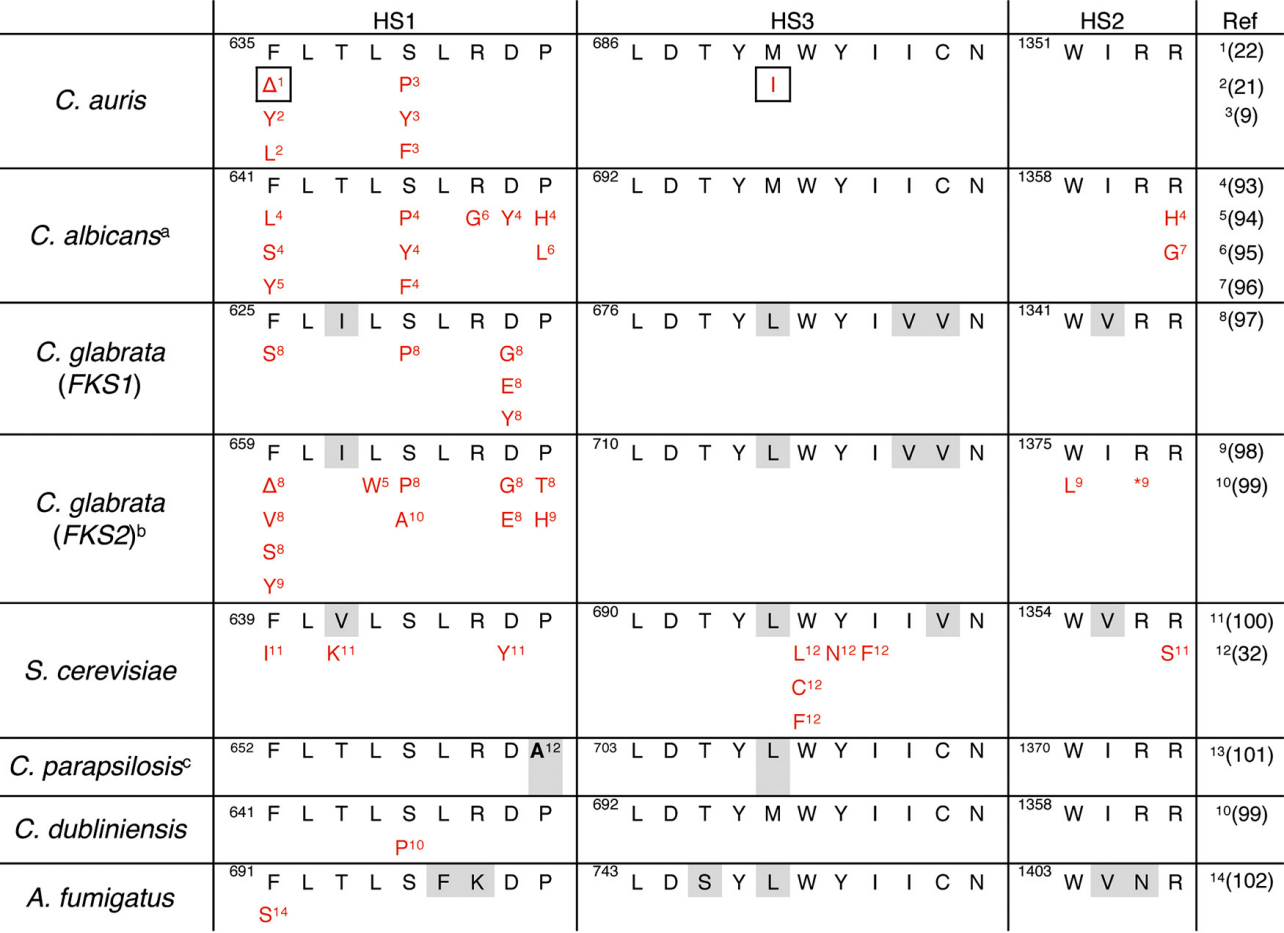
Hot spot (HS) region mutations of the *FKS* genes that confer echinocandin resistance. Amino acid sequence of hot spots 1, 2, and 3 (HS1 to -3, respectively) of C. auris and other fungi are aligned along with all mutations found to decrease echinocandin susceptibility as described in the literature (references are given between brackets). Species-specific polymorphisms of HS are indicated in gray, and the mutations found to confer echinocandin resistance in this study are indicated by a grid. Δ, deletion; *, nonsense mutation; a, mutations R647G and P649L were exclusively heterozygous; b, *FKS2* and *FKS1* are functionally redundant in C. glabrata and both mutated in echinocandin-resistant isolates; c, the naturally occurring alanine at position 660 allows intrinsic reduced echinocandin susceptibility in C. parapsilosis.

Caspofungin resistance was evolved twice in this study, once as monoresistance in the C-lineage and once in the context of multidrug resistance in the FC-lineage, derived from the fluconazole-resistant strain F30 (Fig. 2). The susceptibility to caspofungin decreased drastically in both strain C20 and FC17 (MIC_50_, >64 μg/ml) (Fig. 2). Whole-genome sequencing revealed three mutations in the C20 strain, a missense mutation (atG/atA|M690I) and codon deletion (ttcttg/ttg|FL635L) in *FKS1* (B9J08_000964; Table 1), the gene encoding the catalytic subunit of the echinocandin drug target β(1,3) d-glucan synthase, and one missense mutation (Cta/Ata|L207I) in *ERG3*, encoding sterol Δ5,6-desaturase (B9J08_003737; Table 1). The exact same codon deletion (ttcttg/ttg|FL635L) in *FKS1* emerged independently during caspofungin resistance evolution in the FC-lineage (Table 1). Two additional mutations emerged during the evolution of strain FC17, namely, a missense mutation (gAt/gTt|D367V) in the *PEA2* gene, encoding a subunit of the polarisome (B9J08_001356; Table 1), and a missense mutation (Gca/Aca|A27T) in the *CIS2* gene, encoding a γ-glutamylcysteine synthetase (B9J08_003232; Table 1). Tracing back the emergence of these mutations shows that the *FKS1* mutation FL635L was accompanied by a 500-fold increase in MIC_50_, from 0.125 μg/ml to 64 μg/ml (Fig. 2). The emergence of mutations in *CIS2* (emerged in FC16) and *ERG3* (present in C20) is associated with the further increase of the caspofungin MIC_50_, exceeding 64 μg/ml (Fig. 2). Acquired echinocandin resistance in fungi has been associated with several specific mutations in three defined “hot spot” regions (HS) in the *FKS1* gene (32). Figure 4 shows an amino acid sequence alignment of the *FKS1* gene HS1, HS2, and HS3 regions, constructed to compare the mutations found in this study to those known to confer echinocandin resistance in C. auris and other fungi as described in the literature. This literature review shows that the codon deletion at position F635 as found in this study also has been reported to confer decreased echinocandin susceptibility in Candida glabrata (32) and recently in a C. auris clade I strain (22). The same amino acid was substituted (not deleted as in the C-lineage here) in echinocandin-resistant C. auris strains reported prior (21). The *FKS1* mutation M690I is located in hot spot region 3 without comparable mutations in pathogenic fungi (Fig. 4) and seems to have no direct impact on the drug susceptibility to caspofungin as measured in the C-lineage (Fig. 2).

**FIG 5:**
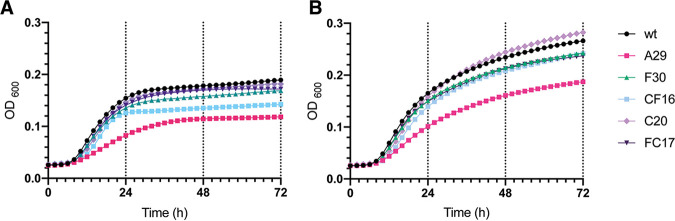
Growth curves of endpoint evolved strains. Growth curves were plotted based on culture density (spectrophotometric quantification of OD_600_; see Materials and Methods) over 72 h of incubation in RPMI-MOPS medium containing (A) 0.2% glucose and (B) 2% glucose at 37°C. Data points are average values of three biological repeats, each represented by the average of two technical repeats.

The mutations in the essential *FKS1* gene do not seem to have a significant effect on the fitness of the strains as shown in growth (Fig. 5), stress tolerance (Fig. S4), and cytotoxicity assays (Fig. S5). Interestingly, strain FC17 (mutations in *FKS1* and *CIS2*, see Fig. 2) shows an increased cytotoxicity toward HeLa cells compared to all other strains (Fig. S5).

10.1128/mBio.03333-20.4 FIG S4 Stress tolerance of evolved strains. To assess fitness, survival/growth of evolved strains was assessed in different conditions of temperature (A), oxidative stress (B), membrane stress (C), osmotic stress (D), and pH stress (E), as described in Materials and Methods. Only 1 of 3 biological repeats and 9 technical repeats is shown, as all repeats showed similar results. Stress conditions without any growth or with no differential growth between the strains are not shown. Download FIG S4, TIF file, 1.1 MB. Copyright © 2021 Carolus et al. 2021 Carolus et al. https://creativecommons.org/licenses/by/4.0/ This content is distributed under the terms of the Creative Commons Attribution 4.0 International license.

10.1128/mBio.03333-20.5 FIG S5 Cytotoxicity of evolved strains. The relative cytotoxicity of the endpoint evolved strains toward HeLa cells after 24 h (A) and 72 h (B) was evaluated using an LDH cytotoxicity assay as described in Materials and Methods. Bars represent means with standard deviation accounting for data obtained from 3 biological repeats, each represented by the mean of 3 technical repeats. Asterisks indicate a significant difference in relative cytotoxicity; *, *P* ≤ 0.05; **, *P* ≤ 0.01; ***, *P* ≤ 0.001; ****, *P* ≤ 0.0001. Download FIG S5, TIF file, 0.03 MB. Copyright © 2021 Carolus et al. 2021 Carolus et al. https://creativecommons.org/licenses/by/4.0/ This content is distributed under the terms of the Creative Commons Attribution 4.0 International license.

The high febrile temperature mimicking condition (growth at 42°C, see Fig. S4A) is the only condition for which strain C20 seems to slightly differ from the wt strain. This phenotype is also present in strain CF16, of which strain C20 is the progenitor (Fig. 1B). Remarkably, the growth curve of strain C20 shows higher optical density (OD_600_) values measured after 36 h in RPMI-MOPS medium supplemented with 2% glucose compared to the wt strain (Fig. 5B). Superior growth of C20 is not observed under the physiological condition of RPMI-MOPS medium supplemented with 0.2% glucose (Fig. 5A). Overall, the fitness assessment shows no significance trade-offs associated with the mutations in *FKS1* and *ERG3* of strain C20.

### Cross-resistance to amphotericin B and fluconazole was established after mutagenesis of *ERG3*, *ERG11*, *FLO8*, and *MEC3* and seems to be accompanied with fitness trade-offs.

During microevolution, the MIC of amphotericin B increased 8-fold in the A-lineage, from an MIC_50_ of 0.5 μg/ml (wt strain) to an MIC_50_ of 4 μg/ml (strain A29; Fig. 2). Simultaneously, cross-resistance to fluconazole emerged, with an MIC increase from 1 μg/ml to over 64 μg/ml (Fig. 2). Two nonsense mutations in genes involved in the ergosterol biosynthesis pathway were discovered (Table 1), namely, the tgG/tgA|W182* mutation in the *ERG3* gene and the Gag/Tag|E429* mutation in the *ERG11* gene, encoding lanosterol 14-alpha-demethylase (B9J08_001448; Table 1). The *ERG11* mutation of strain A29 lies within a region of *ERG11* that corresponds to a frequently mutated (“hot spot”) region of *ERG11* in azole-resistant C. albicans (33, 34). It is, however, distinct from the three SNPs of *ERG11* (namely, Y132F, K143R, and F126L) that have been linked to drug (azole) resistance in C. auris so far (7–9, 14) and are situated in another hot spot region of *ERG11* (33, 34).

**FIG 6:**
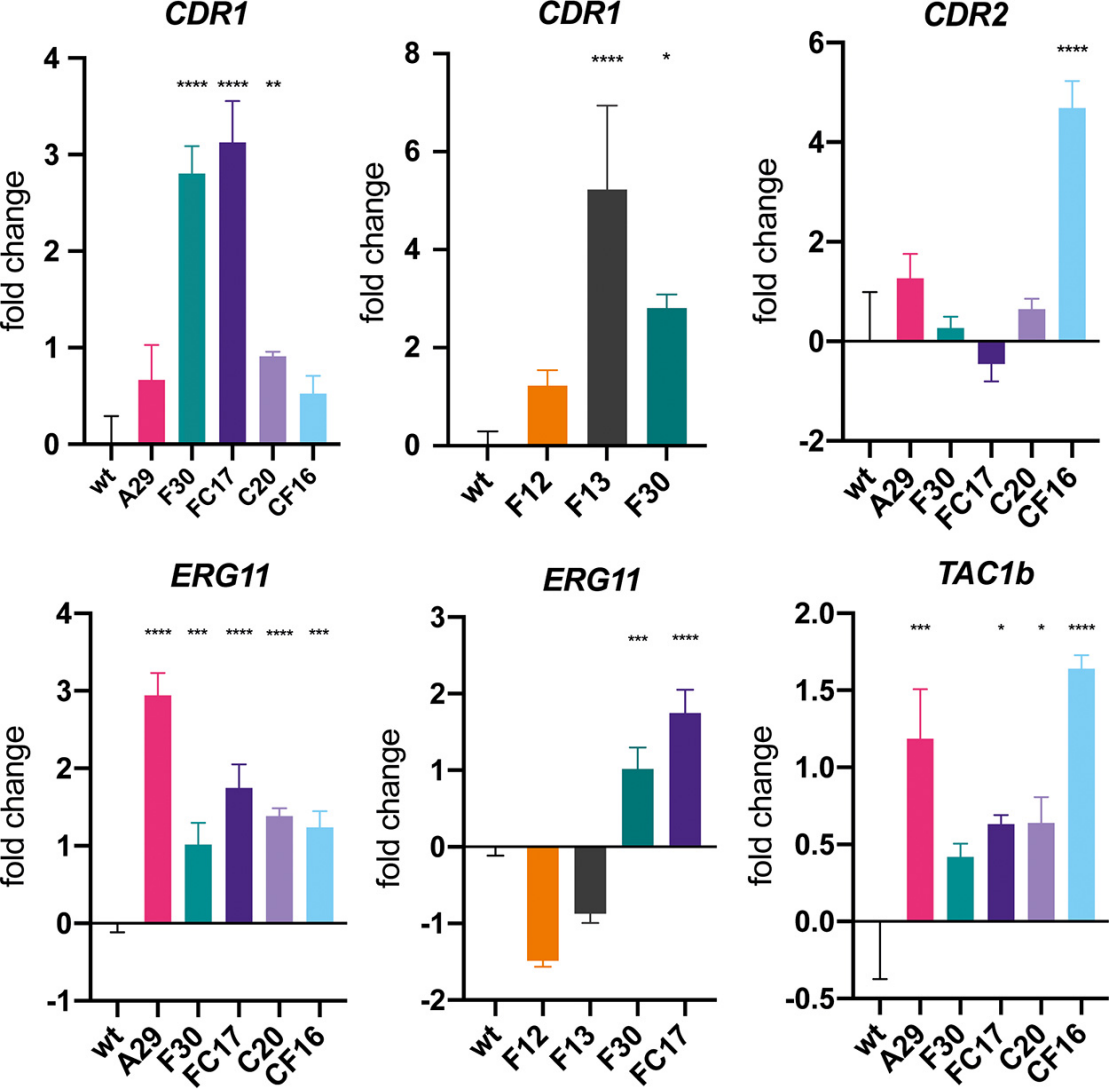
Relative expression of various genes of interest among evolved strains. Fold change of expression levels for *CDR1*, *CDR2*, *ERG11*, and *TAC1b* for the wild type (wt), endpoint evolved strains (A29, F30, FC17, C20, CF16) and intermediate strains F12 and F13 (for *CDR1* and *ERG11*). Bars represent log_2_-transformed means with standard deviation accounting for data obtained from 3 biological repeats, each represented by the mean of 2 technical repeats. Asterisks indicate significant overexpression; *, *P* ≤ 0.05; **, *P* ≤ 0.01; ***, *P* ≤ 0.001; ****, *P* ≤ 0.0001.

Additionally, a nonsense mutation (Cag/Tag|Q384*) was found in the transcription factor encoding the *FLO8* gene (B9J08_000401; Table 1), and a missense mutation (gCg/gTg|A272V) emerged in the *MEC3* gene which encodes a subunit of the Rad17p-Mec3p-Ddc1p sliding clamp (B9J08_003102; Table 1). Remarkably, the mutation in *MEC3* is accompanied by a 2-fold decrease in amphotericin B susceptibility (from an MIC_50_ of 2 μg/ml in strain A21 to an MIC_50_ of 4 μg/ml in strain A27; see Fig. 2). The mutation in *FLO8* did not seem to alter the drug susceptibility for fluconazole or amphotericin B for the concentrations tested. Additionally, strain A29 was found to significantly overexpress *TAC1b* and *ERG11*, as shown by reverse transcriptase quantitative PCR (RT-qPCR), depicted in Fig. 6. Characterization of the growth (Fig. 5), stress tolerance (Fig. S4), and cytotoxicity (Fig. S5) of strain A29 and the wt strain shows, however, that fitness trade-offs accompany the accumulation of the four above-mentioned mutations. Strain A29 shows a lower growth rate in exponential phase and a lower density at stationary phase than the wt strain (Fig. 5), especially in glucose-limiting conditions (Fig. 5A). This growth defect might explain the significantly reduced cytotoxicity towards HeLa cells in this strain (Fig. S5). The spot assays displayed in Fig. S4 show that this strain shows a reduced tolerance to high temperature (Fig. S4.A), membrane stress (Fig. S4.C), osmotic stress (Fig. S4.D), and low pH (Fig. S4.E).

### Fluconazole resistance is linked to two aneuploidies and a mutation in transcription factor *TAC1b*.

In strain F13, a codon deletion (ttc/|F15) in the *TAC1b* gene was identified (B9J08_004820; Table 1) that corresponds to a 32-fold increase in the MIC_50_ of fluconazole (Fig. 2). Tac1b is a transcription factor that positively regulates the expression of the ATP binding cassette (ABC) transporter Cdr1, known to be involved in azole efflux and azole resistance in C. auris (16, 26). Gene expression analysis of strain F12 (no *TAC1b* mutation) and strain F13 (*TAC1b* mutation obtained) confirms that the acquisition of this mutation corresponds to a significantly increased expression of *CDR1* (B9J08_000164), as shown in Fig. 6. The overexpression of *CDR1* is maintained in strain F30 and in the multidrug-resistant strain FC17, as shown in Fig. 6. Although the mutation in strain F13 is novel to C. auris, it is located in a region of *TAC1b* that is known to harbor gain-of-function mutations in fluconazole-resistant clinical isolates of C. auris, as shown by Rybak et al. (16).

Two aneuploidies independently emerged during fluconazole resistance evolution. Read coverage of whole-genome sequencing was used to analyze copy number variation (CNV) by calculating the normalized depth read coverage per 5 kb window (see Materials and Methods). A visual representation of this normalized coverage for each chromosome in all endpoint sequenced strains is displayed in Fig. 3. This reveals that a segmental and whole-chromosome duplication emerged in the F- and CF-lineages, respectively.

The 191-kb segmental duplication of Chr1 in the F30 strain contained 75 protein-encoding genes (based on the B11220 reference genome annotation; GenBank accession numbers CP043531 to CP043537), including *ERG11*. During further evolution to caspofungin resistance in the FC-lineage (see Fig. 1B), this segmental duplication was maintained but decreased in size to 161 kb containing 67 protein-encoding genes (still including *ERG11*). The segmental Chr1 duplication is the only genetic difference between strain F13 and strain F30 that can be attributed to the increase in MIC_50_ of 32 μg/ml (in strain F13) to a MIC_50_ of >64 μg/ml (in strain F30), as shown in Fig. 2. Expression analysis showed that the duplication in strain F30 is accompanied by an increased expression of *ERG11*, not present in strain F13 (Fig. 6). Strain F30 was also marked by a slight decrease in amphotericin B susceptibility, retained in the FC17 strain (Fig. 2). This is possibly due to *ERG11* overexpression.

The whole chromosome 5 (Chr5) duplication in the CF16 strain contained a region of 933 kb encompassing 405 protein-encoding genes, including *TAC1b*. This aneuploidy marks the only genetic difference between strain C20 and strain CF16 and is therefore suggested to confer the 32-fold decrease in fluconazole susceptibility between those strains (Fig. 2). Expression analysis showed that the duplication of Chr5 correlates with a significant overexpression of *TAC1b* and *CDR2* (B9J08_002451), but not *CDR1* in strain CF16 (Fig. 6).

The mutation in TAC1b and segmental duplication of Chr1 did not seem to have any major effect on the growth (Fig. 5), stress tolerance (Fig. S4), and cytotoxicity (Fig. S5) of strain F30 compared to the wt strain. The chromosome 5 duplication might, however, drive the slight reduction in growth at stationary phase (Fig. 5) and tolerance toward certain stressors in strain CF16 compared to its progenitor (C20) and the wt strain (Fig. S4).

## DISCUSSION

### This study shows the evolution of multiple mechanisms known to be involved in drug resistance in fungi, albeit new to C. auris.

In the largest screening of C. auris clade II strains, 62.3% of a total of 61 isolates proved to be fluconazole resistant, although only 3 isolates harbored a known azole-conferring mutation in *ERG11* (K143R) (35). This indicates that other mechanisms of azole resistance play a role in C. auris clade II (35). Here, we suggest that at least four molecular mechanisms, none of which include the most common mutations in *ERG11*, might underlie the decreased fluconazole susceptibility in clade II C. auris. Previous reports show that many GOF mutations in *TAC1* or homologues of this transcription factor can confer azole resistance in *Candida* species (36–38), including C. auris (16), through an overexpression of the drug efflux pump Cdr1. Many GOF mutations are found in the region encoding the putative transcriptional activation domain of *TAC1*, situated in the C-terminal portion of the protein in *Candida* (39). Rybak et al. report one mutation in this region (codon deletion at position F862) to be associated with fluconazole resistance in C. auris, although all other reported resistance-associated mutations lie between the DNA binding, transcription factor, and activation domain of *TAC1b* (16). One such mutation (F214S), discovered in an experimentally evolved strain of C. auris (16), lies in the proximity of the codon deletion at position 191 as we discover here. Based on these reports and our findings, we therefore hypothesize that F191Δ is a new potential gain of function mutation of C. auris *TAC1b*, conferring azole resistance through *CDR1* overexpression. Nevertheless, previous reports have shown that Tac1b might function in other, Cdr1-independent ways to decrease azole susceptibility in C. auris (16, 26). Another genetic adaptation linked to reduced azole susceptibility in this study involves aneuploidies. In C. albicans, both *TAC1* and *ERG11* are located on Chr5, and the duplication of this region by forming an isochromosome [i(5L)] has been reported to confer azole resistance *in vitro* and *in vivo* (40). Based on this, and other reports on azole resistance in C. auris due to CNVs and/or overexpression of *ERG11* (9, 15, 41), we hypothesize that a similar mode of action is at play in strain F30. Moreover, comparing drug susceptibility between strain F13 and F30, the overexpression of *ERG11* in strain F30 more than doubles the fluconazole MIC_50_ compared to—the already resistant—strain F13, while it decreases the susceptibly for amphotericin B (Fig. 2). Recently, azole resistance in an experimentally evolved C. auris clade I strain was linked to the duplication of chromosome 5 and consequent upregulation of *ERG11* (Chr1) and *TAC1b* but not *CDR1* (Chr5) (19). In this study, the duplication of C. auris Chr5 is the only genomic alteration that distinguishes strain CF16 from strain C20, and therefore, we propose that this duplication is, here too, responsible for azole resistance. Expression analysis shows that the duplication and subsequent overexpression of *TAC1b* (Fig. 6) does not correspond to an increased expression of *CDR1*, making our findings consistent with the report of Bing et al. (19). *TAC1b* may play a CDR1-independent role in azole resistance of C. auris, as suggested by Mayr et al. (26), and since *CDR2* is significantly upregulated in strain CF16, its function in azole resistance should be further investigated.

The acquisition of resistance to polyenes is among the least understood of all antifungal drugs and has been linked to mutations in genes involved in the ergosterol biosynthesis pathway in *Candida* spp., including alterations in *ERG2* (42), *ERG6* (43), *ERG11* (44), and *ERG3* (45). Cross-resistance to azoles and amphotericin B has often been associated with the abrogation of two *ERG* genes simultaneously (46). One such example is the combination of the loss of function (LOF) of *ERG11* and *ERG3* in C. tropicalis (47, 48). Upon the abrogation of *ERG11*, due to a LOF mutation or the action of azoles, a toxic 3,6-diol derivative is produced through the action of the sterol Δ^5,6^ desaturase, encoded by *ERG3* (49). Simultaneous disruption of the function of both *ERG3* and *ERG11* can prevent this detrimental effect (46). Here, we hypothesize that such a mechanism of cross-resistance might be established also in C. auris. The fact that all clones tested (over 60) between generation A20 and A21 had either both or no nonsense mutations can be explained by simultaneous emergence or an epistatic effect (i.e., the effect of a mutation in one gene depends on a mutation in another gene) of *ERG3* and *ERG11 LOF* mutations in strain A21 (Fig. 2). In the latter scenario, the AmB conferring nonsense mutation in *ERG11* was only possible in an *ERG3-*LOF background and both emerged in a quick and dependent fashion.

Target alteration is the most commonly observed and most studied mechanism of echinocandin resistance in *Candida* species (50). In C. auris, most echinocandin resistance-conferring *FKS1* mutations occur at position S639 (12, 20). Most recently, however, a SNP (21) and codon deletion (22) of *FKS1* F635, the same codon deleted in strain C3 in this study, was linked to reduced echinocandin susceptibility of C. auris in the clinic. In general, mutations in echinocandin-resistant *Candida* species lie within two small, strictly defined hot spot regions of *FKS1* (50). However, the codon substitution at position 690 that emerged in strain C15, occurs in the elusive hot spot 3, a third potent hot spot region discovered by site-directed mutagenesis of *FKS1* in Saccharomyces cerevisiae (32). This mutation occurred after the codon deletion at position 635 (in HS1) in the C-lineage but did not affect the echinocandin MIC_50_, possibly indicating functional compensation of the altered Fks1 protein. A third mutation of strain C20 occurred in *ERG3*. One report shows that a mutation in *ERG3* in a clinical Candida parapsilosis strain conferred resistance to both azoles and echinocandins (17). Here, we observe a slight increase, rather than a decrease, in fluconazole susceptibility upon the emergence of the *ERG3* mutation in strain C20 (Fig. 2). Most interestingly this mutation further increases the MIC_50_ for caspofungin in strain C20 compared to strain C15, which only harbored *FKS1* mutations (Fig. 2). Overall, the caspofungin-resistant strains (FC17, C20) obtained in this study show echinocandin MIC values (>64 μg/ml) that significantly exceed any previously reported MIC values for echinocandins in C. auris (8, 51–53). As these mutations evolved rapidly (Fig. 2) and do not seem to greatly impact the overall fitness of these strains (see Fig. 5 and Fig. S4 and S5), they might impose a significant clinical threat. We therefore suggest that the clinical significance of the reported mutations, including the role of *ERG3*, should be further investigated.

### Four genes were mutated that were previously not or vaguely associated with drug resistance in fungi.

*FLO8*, mutated in the amphotericin B-resistant strain A29, encodes a transcription factor known to be essential for filamentation in C. albicans (54). This filamentation was shown to decrease the rate of programmed cell death in C. albicans when exposed to amphotericin B (55). Flo8 has multiple downstream effects, one of which is the positive regulation of *ERG11* expression, shown in S. cerevisiae (56), and thus potentially playing a role in azole and amphotericin B resistance. In a recent study of clinical C. auris isolates from South America, a nonsynonymous mutation in the *FLO8* gene significantly correlated with amphotericin B resistance (25). In a follow-up study on the structure of Flo8, the authors suggest a potential role of Flo8 in C. auris virulence and drug resistance, arguing that the *FLO8* mutation found before (25) could be a gain of function mutation (57). In our study, however, we see a nonsense mutation, abrogating Flo8 at amino acid 100 and thus assumed to be disruptive to its function. Earlier, a LOF of *FLO8* was found to play a role in azole resistance, with a *FLO8* deletion correlated to increased *TAC1*, *CDR1*, and *CDR2* expression, while *FLO8* overexpression led to decreased *CDR1* expression (58). Although these reports strengthen the suspicion of a role of Flo8 in drug resistance, we cannot further explain the influence of the *FLO8* mutation on the resistance phenotype observed here. Further research on Flo8 in drug resistance is therefore highly desirable.

The fourth gene mutated during amphotericin B resistance evolution is an ortholog of *MEC3*, encoding a DNA damage checkpoint protein as part of the Rad17p-Mec3p-Ddc1p sliding clamp, primarily involved in DNA damage recognition and repair in S. cerevisiae (59). No clear reports of a function for *MEC3* in antifungal drug resistance were found, although two studies mention the upregulation of *MEC3* upon the acquisition of azole resistance in an experimentally evolved C. glabrata strain (38, 60). Our results show that the mutation in *MEC3* has a significant influence on susceptibility to amphotericin B (Fig. 2). The mechanism behind increased amphotericin B resistance upon acquiring a mutation in *MEC3* remains unclear, although an altered DNA damage recognition response might prevent apoptosis upon exposure to amphotericin B. Multiple reports concerning the mode of action of polyenes suggest that, besides a pore-forming and sterol adsorption effect, polyenes such as amphotericin B are cytotoxic through oxidative damage, which includes protein carbonylation, lipid peroxidation, and DNA damage (23, 61–65). The latter is recognized by DNA damage checkpoint mechanisms and can lead to apoptosis (66), possibly explaining the phenomenon we observe here. Other reports show that defects in DNA damage recognition might drive the emergence of drug resistance in fungi, as they can increase mutation rates (67–69). Notably, a recent report of resistant C. auris in a hospital in India, shows that all (*n* = 9) sequenced isolates harbored a nonsense mutation in *MSH2*, a DNA mismatch repair gene (24).

Strain FC17 harbored a mutation in *CIS2.* The S. cerevisiae ortholog (*ECM38*) of *CIS2* encodes a γ-glutamyltranspeptidase, involved in glutathione degradation (70), detoxification of xenobiotics (71), and cell wall biogenesis (72). The role that *CIS2* plays in the latter, regarding echinocandin resistance, remains unclear, but a study from Maras and colleagues (73) illustrated that fluconazole and micafungin resistance were accompanied by altered levels of glutathione in C. albicans, hypothesized to counteract oxidative stress caused by these antifungal drugs. Interestingly, strain FC17 shows an oxidative stress tolerance similar to the wt and mono-resistant strains and higher than the other MDR strains (strain A29 and CF16; see Fig. S4B). Additionally, the cytotoxicity assessment shows that strain FC17 has a significantly higher cytotoxic effect than the wt and other strains after 72 h (Fig. S5B**)**. This might be linked to the mutation in *CIS2* and an increased oxidative stress tolerance. Nevertheless, in the study by Maras and colleagues (73), the increased levels of glutathione were accompanied by the overexpression of γ-glutamylcysteine synthetase (73). A role for *CIS2* and glutathione catabolism in drug resistance, mediated by an altered redox metabolism remains to be elucidated.

The fourth mutation in the FC-lineage lies within a gene predicted to encode *PEA2*, a subunit of the polarisome, involved in polarized growth and morphogenesis in S. cerevisiae (74). This mutation has, however, no significant effect on the drug resistance profile and might thus be the result of random genetic drift.

### Experimental evolution can be a powerful tool to research resistance, although it has limitations.

Due to recent advances in next-generation sequencing technology, genome-wide studies of drug resistance have become more common (75, 76). The classic approach of sequencing drug-resistant clinical isolates directly from patients (75) has many limitations, including the frequent unavailability of the original drug-susceptible genotype and the difficulty in resolving mutations associated with drug resistance from those that have accumulated due to host-pathogen interactions and random genetic drift. *In vitro* experimental evolution copes with most of these problems (75, 77), is highly repeatable, and allows controlled long-term monitoring of different strains and conditions. Moreover, the ability to isolate and investigate each generation separately allows monitoring of both the speed and the stepwise progression of drug resistance development. Nevertheless, *in vitro* experimental evolution has its own limitations, such as the homogeneity of the selective pressure in the absence of metabolization of the drug, tissue-specific exposure, and host-pathogen interactions. However, studies of drug resistance by *in vitro* evolution often resemble acquired resistance found in clinical isolates (13, 16, 75, 77, 78). In regard to our results, a comparative analysis of mutations reported in the literature and reanalysis of variants predicted in 304 sequenced clinical isolates of C. auris (9) show that most mutations reported here are novel to this species. Although the *in vitro* context in which these mutations evolved might explain this, one must be careful by regarding these findings to be nonrelevant to the *in vivo* setting or clinical environment. Reports on resistance mutations (providing whole-genome analysis) are still scarce and the database of 304 sequenced clinical isolates of C. auris (9) is limited, with only 23% of isolates reported being multidrug-resistant and only 7 clinical isolates belonging to clade II (6 isolates pan-susceptible, 1 isolate fluconazole-resistant) (9). This study and other studies of bacteria (79) and fungi (13, 77, 80) show that *in vitro* experimental evolution can be a powerful tool, especially if combined with an effective approach to trace the full evolutionary history of mutation events, as we did here using allele-specific PCR. Nevertheless, we need to point out that the preferred way of validating molecular mechanisms for resistance should rely on gene-editing the evolved mutations in the parental (wild type) background and/or editing mutant alleles to the wild type form in evolved strains. For this, a CRISPR-based gene editing system would be ideal. So far, no scarless allele-specific CRISPR gene editing system in C. auris has been optimized or reported, although alleles have been deleted, replaced, and complemented with an inducible promoter (16, 18, 27). The CRISPR system reported by Kim et al. (18), based on a system for C. albicans (81), could be used for allele editing in C. auris, but it makes use of the stable integration of the CRISPR cassette into the *ENO1* locus (81). Disruption of the *ENO1* locus in C. albicans has been reported to alter growth, virulence, and drug susceptibility (82). If the same phenotypes would apply to C. auris, it would complicate the interpretation of the effect that introduced mutations have in the GMO strain. Moreover, constitutive *cas9* expression can have off-target effects (83), including an effect on the fitness of the transformed cells, as reported for C. glabrata (84). In conclusion, neutral gene editing, which preferably relies on transient expression of *cas9*, is a highly desired tool for future molecular research in C. auris and should be regarded as a priority in the field.

## MATERIALS AND METHODS

### Strains and growth conditions.

All experiments were performed with C. auris strain B11220 (CBS10913) from the Westerdijk Fungal Biodiversity Center (wi.knaw.nl/). Strains were grown on yeast extract-peptone-dextrose (YPD) agar (2% glucose) at 37°C and enriched in RPMI 1640 (Thermo Fisher Scientific)-MOPS (morpholinepropanesulfonic acid) liquid medium containing 2% glucose at 37°C in a shaking incubator overnight. All strains, including daily aliquots of serially transferred populations in the microevolution assay, were stored at −80°C in RPMI-MOPS medium containing 25% glycerol.

### Antifungal susceptibility testing.

The MIC was determined using a broth dilution assay (BDA) based on Clinical and Laboratory Standards Institute (CLSI) guidelines (85). In short, a dilution of 64 μg/ml to 0.06 μg/ml of each drug was prepared in RPMI-MOPS medium in a 96-well polystyrene microtiter plate. A standardized amount of 100 to 500 cells was dissolved in a final volume of 200 μl per well, and plates were incubated at 37°C. Growth was measured after 48 h of incubation through spectrophotometric quantification of the OD_600_ in a SPECTRAmax Plus 384 microplate reader (Molecular Devices). Resistance was determined through tentative breakpoints provided by the CDC (6).

### *In vitro* experimental evolution assay.

An overview of the design of the experimental evolution assay is given in Fig. 1A. At the start of the evolution experiment, 10^6^ cells are diluted in a 5 ml volume of RPMI-MOPS medium (2% glucose) containing no drug (control) or a drug at a concentration of 0.5× MIC_50_, 1× MIC_50_, or 2× MIC_50_. All conditions were performed in triplicate (3 evolving populations per condition). After 24 h of incubation at 37°C in a shaking incubator, growth of each population was compared to the average growth of 3 controls (no drug) by spectrophotometric quantification (OD_600_). Next, 500 μl of each population was transferred to 4,500 μl of fresh medium with a concentration of drug equal to the previous culture when OD_600_ (evolving population) ≤ OD_600_ (average control) or double compared to the previous culture when OD_600_ (evolving population) > OD_600_ (average control). The experiment was terminated after 30 days or if the MIC_50_ exceeded the resistance breakpoint value, as evaluated by intermediate MIC testing (using BDA). At the end of each evolution experiment, a single colony was picked as the progenitor for a consecutive evolution experiment.

### Analysis of growth.

Growth was assessed by spectrophotometric observation (OD_600_) over time in a Multiskan GO (Thermo Scientific) automated plate reader using flat-bottom 96-well plates and intermittent (30 min interval) pulsed shaking (medium strength, 5 min). Cultures were diluted in RPMI-MOPS medium containing 0.2% or 2% glucose, to a final volume of 10^6^ cells per well. Growth was measured for 72 h at 37°C. Growth curves were plotted as an average value of 3 biological repeats with 3 technical repeats per biological repeat.

### Analysis of stress tolerance.

The survival of C. auris cells in various degrees of temperature-, oxidative-, membrane-, osmotic- and pH-induced stress was assessed using spot assays. C. auris cells were resuspended in phosphate-buffered saline (PBS) at concentrations of 10^7^, 10^6^, 10^5^, and 10^4^ cells/ml and 1 μl of each dilution was spotted on YPD agar containing 2% glucose. Plates were incubated for 48 h at 37°C or at 30°C, 37°C, and 42°C for the evaluation of temperature stress. Oxidative stress was evaluated on agar containing 6, 8, or 10 mM H_2_O_2_ (Sigma-Aldrich). Membrane stress was evaluated on agar containing 0.005, 0.05, or 0.2% sodium dodecyl sulphate (SDS; Sigma-Aldrich). Osmotic stress was evaluated on agar containing 1, 1.75, or 2.5 M NaCl (Sigma-Aldrich). pH stress was evaluated on agar adjusted to pH 4, 5, 6, or 7 by supplementing the agar with HCl or KOH. Agar adjusted to pH 4 and 5 was buffered with 50 mM citrate buffer, while agar adjusted to pH 6 was buffered with 50 mM morpholineethanesulfonic acid (MES) buffer, and agar adjusted to pH 7 was buffered with 100 mM MOPS buffer. All spot assays were performed on 3 biological repeats with 3 technical repeats per biological repeat, of which one example is shown.

### Analysis of relative cytotoxicity.

HeLa cells (Thermo Fisher Scientific) were cultured in complete Dulbecco’s modified Eagle medium (DMEM) at 37°C and 5% CO_2_, using 25-cm^2^ polystyrene culture flasks. Upon reaching 80% confluence, cells were washed twice with PBS, trypsinized using 0.5% trypsin EDTA (Gibco), and replenished to allow cell adhesion in polystyrene 96-well plates as a 100-μl suspension of 10^5^ cells/ml fresh DMEM culture medium for 24 h, at 37°C and 5% CO_2_. A 10 μl volume of 10^5^ C. auris cells or PBS (negative control) was added to the HeLa cells, followed by incubation at 37°C and 5% CO_2_ for 24 h or 72 h. Cytotoxicity of C. auris toward HeLa cells was assessed using a lactate dehydrogenase (LDH) cytotoxicity assay as follows: 10 μl of cell lysis buffer (Invitrogen) was added to cultures of HeLa cells with C. auris or pure HeLa cells (positive control), followed by incubation for 45 min at 37°C and 5% CO_2_. Then, 50 μl of supernatant (lysate) was mixed with 50 μl of substrate mix (Invitrogen) in a separate 96-well plate and incubated for 30 min at room temperature in darkness. Next, 50 μl of stop solution (Invitrogen) was added, followed by spectrophotometric quantification of the OD_490_ and OD_680_ in a Synergy H1 hybrid plate reader (BioTek). LDH activity was calculated by subtracting the OD_680_ from the OD_490_, and relative toxicity was calculated as $\text{Cytotoxicity }\%\;=\frac{\mathrm{LDH}\;\mathrm{activity}\;\mathrm{of}\;\mathrm{treated}\;\mathrm{cells}\;-\;\mathrm{LDH}\;\mathrm{activity}\;\mathrm{of}\;\mathrm{negative}\;\mathrm{control}}{\mathrm{LDH}\;\mathrm{activity}\;\mathrm{of}\;\mathrm{positive}\;\mathrm{control}\;-\;\mathrm{LDH}\;\mathrm{activity}\;\mathrm{of}\;\mathrm{negative}\;\mathrm{control}}\times 100$. Relative cytotoxicity for each strain was measured for 3 biological repeats, each represented by the average of 3 technical repeats, and compared statistically with GraphPad Prism using a one-way analysis of variance (ANOVA) with multiple comparisons in respect to the wt strain.

### DNA extraction.

Genomic DNA for whole-genome sequencing was extracted using the MasterPure yeast DNA purification kit (Lucigen, USA) following the manufacturer’s protocol. For (AS-)PCR and Sanger sequencing, DNA was isolated from the cells through phenol chloroform isoamyl alcohol (PCI) extraction. Cells were dissolved in 300 μl Tris EDTA (TE) buffer with 300 μl PCI solution (phenol pH 6.7, chloroform, and isoamylalcohol at 25:24:1) and lysed by microbead shearing in a FastPrep-24^TM^ Classic lysis system (20 sec, 6m/sec) (MP Biomedicals). After cell lysis, DNA was isolated and purified using ethanol precipitation. The resulting DNA was diluted to a concentration of 200 ng/μl in milliQ H_2_O, based on the DNA concentration measured through absorbance at 260 nm with a NanoDrop spectrophotometer (Isogen).

### Whole-genome sequencing and analysis.

Genomic libraries were created using the NEBNext Ultra DNA library prep kit for Illumina sequencing (New England Biolabs, USA), and genomes were sequenced on an Illumina MiSeq v2 500 instrument (Illumina, USA), obtaining a coverage of at least 50×. Standard quality control was performed using FastQC v0.11.7 (86). Paired-end reads were aligned using the Burrows-Wheeler Aligner MEM algorithm BWA-MEM v0.7.17 (87) to the annotated genome assemblies of strain B8441 (clade I; GenBank accession number GCA_002759435.2 [15]) and B11220 (clade II; GenBank accession number CP043531 to CP043537 [29]). For SNP and indel identification, the assembly alignment to the annotated genome of strain B8441 (clade I) was used, while CNV analysis was performed using the assembly alignment to reference genome B11220 (clade II), respectively. The genome sequences of all endpoint experimentally evolved strains were deposited in the NCBI Sequence Read Archive (SRA) under BioProject PRJNA664007. Variants were identified and filtered using GATK v4.1.2.1 (88, 89), with the haploid mode, including GATK tools HaplotypeCaller and variant filtration using “QD < 2.0 ‖ FS > 60.0 ‖ MQ < 40.0”. In addition, variants were filtered if they had a minimum genotype (GT) quality of <50, alternate allele frequency of <0.8, or allelic depth (DP) of <10. The final variant call format (VCF) was annotated using SnpEff v4.3T (90). CNVs were identified using CNVnator v0.3 (91), selecting for 1 kb genomic windows of significant (*P < *0.01) variation in normalized coverage. The average depth per 5 kb window was normalized to the coverage of the whole-genome sequence for each isolate and plotted in R (92). Candidate variants were compared with a set of 304 globally distributed C. auris isolates representing clades I, II, III, and IV (9).

### PCR and Sanger sequencing.

Primers for PCR and Sanger sequencing were designed *in silico* using CLC Genomics Workbench v20.0.3. Primer design was based on B11220 whole-genome sequencing (WGS) consensus sequences (GenBank accession number CP043531 to CP043537 [29]) of the regions of interest and sequences of the genes of interest in reference genome C. auris B8441 downloaded from the *Candida* genome database (candidagenome.org). Sequencing primers were designed to include a ±1,000-bp region of interest (spanning the region with the mutation of interest). All primers are given in Table S1.

Amplification of regions of interest was achieved through PCR using Q5 high-fidelity DNA polymerase (New England Biolabs, Inc.). The total reaction volume of 50 μl consisted of 200 ng/μl DNA extract, 5 μl deoxynucleoside triphosphate (dNTP) (0.2 mM), 10 μl Q5 buffer, 0.5 μl Q5 polymerase (2 units), milliQ, and 0.4 μl of both forward and reverse primer (1 μM). The PCR program consisted of initial denaturation at 98°C for 3 sec, 30 cycles of 98°C for 15 sec, 56°C for 25 sec, 72°C for 2 min, and a final elongation step at 72°C for 2 min in a Labcycler Basic thermocycler (Bioké). Correct amplification was verified by performing electrophoresis on a 1% agarose gel at 135 V for 25 min. After verification, the sequencing primers (10 μM) were added to PCR amplicons, and the DNA was sequenced using Sanger sequencing by Eurofins (GATC, Germany).

### Allele-specific PCR (AS-PCR).

The emergence of SNPs and indels was traced back in whole populations and a maximum of 30 single clones (colonies) per population using a rapid sequencing-free method: allele-specific SNP-PCR. Two primer pairs per gene of interest were designed according to Liu et al. (31), consisting of one universal primer and/or one mutant-allele primer or wild-type allele primer, respectively. Primers consist of an allele-specific region at the 3′ terminal nucleotide of the mutant or wild-type allele-specific primer. Additionally, a mismatch at the 3rd nucleotide from the 3′ terminal was included to increase annealing specificity at a wider temperature range (31). All primers used for AS-PCR are listed in Table S1.

To validate primer specificity, a temperature gradient PCR was performed in which the annealing temperature varied between 60°C and 70°C. AS-PCR sensitivity was assessed by performing PCR on serial dilution of the reference DNA template. All PCRs were performed in a total reaction volume of 20 μl consisting of 1 μl of 1/20 dilution of the pure PCI DNA extract, 5 μl dNTP (0.2 mM), 10 μl TaqE buffer, 0.5 μl TaqE polymerase (2 units), milliQ, and 0.4 μl of both forward and reverse primer (1 μM). The PCR program consisted of initial denaturation at 98°C for 3 sec, 30 cycles of 98°C for 15 sec, 25 sec annealing at 61°C (for primers of CIS2, PEA2, TAC1b, FKS1^FL635L^, ERG3^W182*^, and ERG11) or at 63°C (for primers of ERG3^L207I^, FKS1^M690I^, FLO8, and MEC3), 72°C for 2 min, and a final elongation step at 72°C for 2 min in a Labcycler Basic thermocycler (Bioké). Amplification and thus the presence or absence of a mutation were verified by performing electrophoresis on a 1% agarose gel at 135 V for 25 min.

### Gene expression and copy number variation analysis.

C. auris cells from a single colony grown overnight on YPD agar (2% glucose) were enriched in RPMI-MOPS (2% glucose) medium for 16 h. These cultures were diluted to 10^8^ cells in a volume of 50 ml fresh RPMI-MOPS (2% glucose) medium and incubated for 8 h at 37°C in a shaking incubator to ensure the harvested cells were growing in the exponential growth phase. Next, cells were harvested by centrifugation, washing in ice-cold PBS, and snap-freezing in liquid nitrogen to store at −80°C.

For gene expression analysis (RNA extraction and RT-qPCR), cells were resuspended in 1 ml TRIzol (Thermo Fisher Scientific) and lysed by microbead shearing in FastPrep-24^TM^ Classic lysis system (20 sec, 6m/sec) (MP Biomedicals). Nucleotides were extracted by washing the lysate supernatant with chloroform (360 μl) and isopropanol (350 μl) and precipitated by washing three times with 70% ethanol. Nucleotide concentrations and purity were measured spectrophotometrically using a NanoDrop ND-1000 instrument (Isogen Life Science). Extracts were diluted to 1 μg pure nucleotide concentration and purified by DNase treatment (New England Biolabs). cDNA was synthesized from RNA by using the iScript cDNA synthesis kit (Bio-Rad) according to the manufacturer’s recommendations. Real-time qPCR was performed using GoTaq polymerase (Promega) and the StepOnePlus real-time PCR thermocycler (Thermo Fisher) as follows: activation at 95°C for 2 min, 40 cycles of denaturation at 95°C for 3 sec, and annealing/extension at 60°C for 30 sec. Primers used for qPCR were designed with the PrimerQuest tool of IDT and are listed in Table S1. A total of 8 housekeeping genes involved in various cellular processes were assessed, of which the 3 most stable candidates were used in the analysis (i.e., *ACT1*, *LSC2*, *UBC4*). Gene expression analysis was performed using qBasePlus software. Fold change (with standard deviation [SD]) was plotted from log_2_(Y) transformed data and compared statistically (using a one-way ANOVA with multiple comparisons in respect to wt) with GraphPad Prism. Expression analysis in each strain was performed using 3 biological repeats each represented by the average of 2 technical repeats.

For copy number variation analysis, gDNA was extracted as described in “DNA Extraction,” above, and standardized concentrations of 0.5 ng/μl of gDNA were used to quantify target markers (*TAC1b* and *ERG11*) by qPCR using the same protocol and analysis as described above.
