# Supplementary figures and images for: Genome-Wide Analysis of Experimentally Evolved Candida auris Reveals Multiple Novel Mechanisms of Multidrug Resistance
Source: mBio. 2021 Apr 5;12(2):e03333-20. doi: 10.1128/mBio.03333-20 (PMC8092288; doi:10.1128/mBio.03333-20)

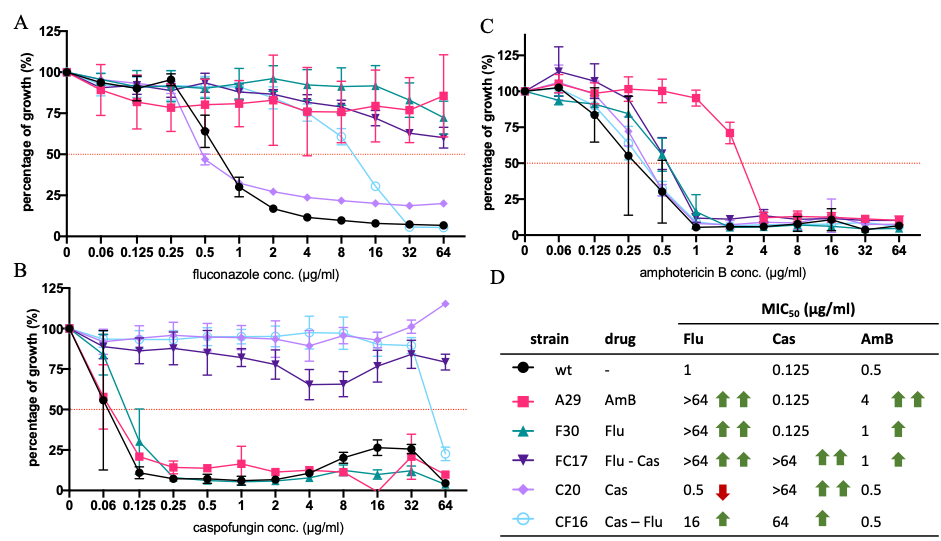

Supplement: FIG S1 [file mBio.03333-20-sf001.tif]

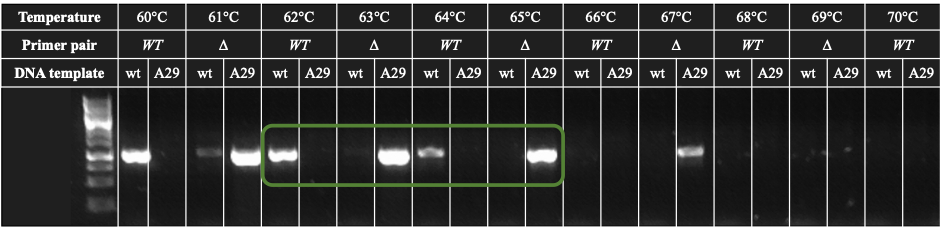

Supplement: FIG S2 [file mBio.03333-20-sf002.tif]

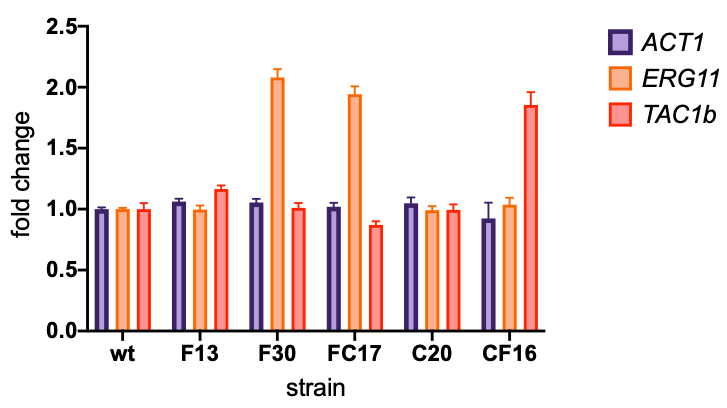

Supplement: FIG S3 [file mBio.03333-20-sf003.tif]

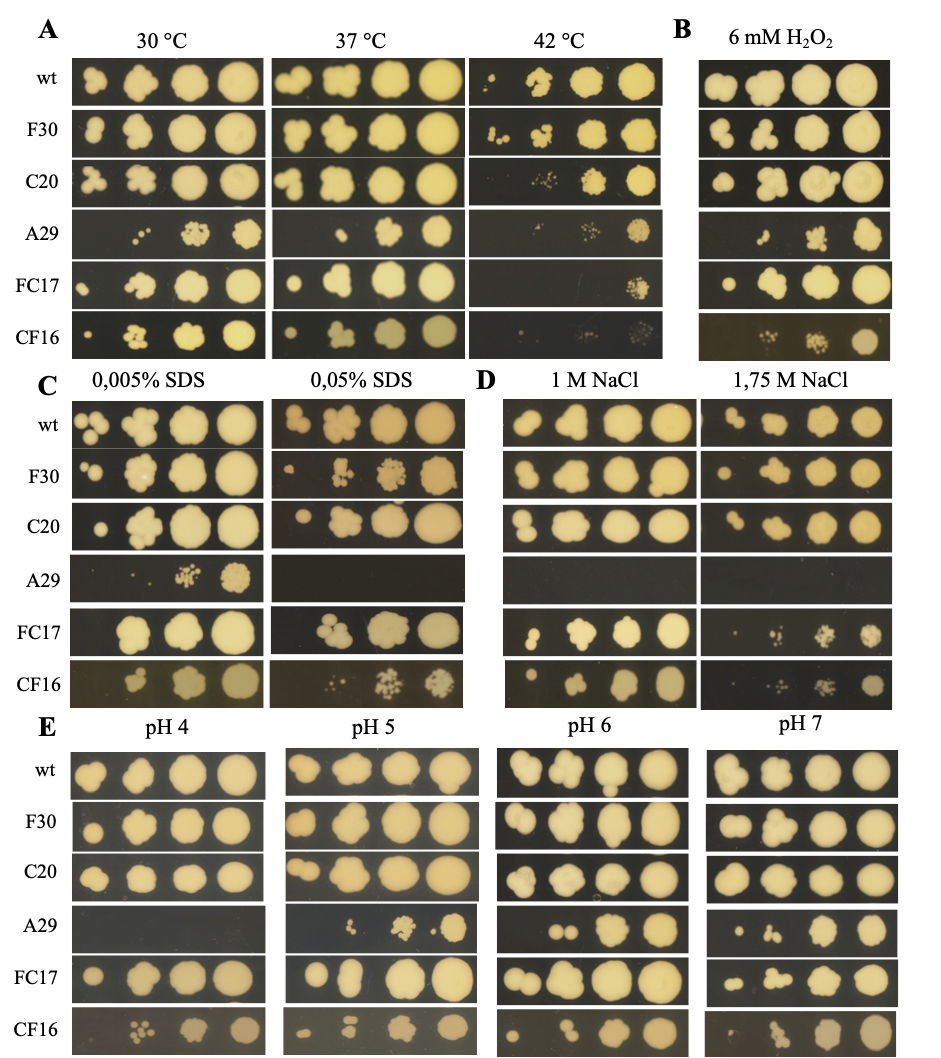

Supplement: FIG S4 [file mBio.03333-20-sf004.tif]

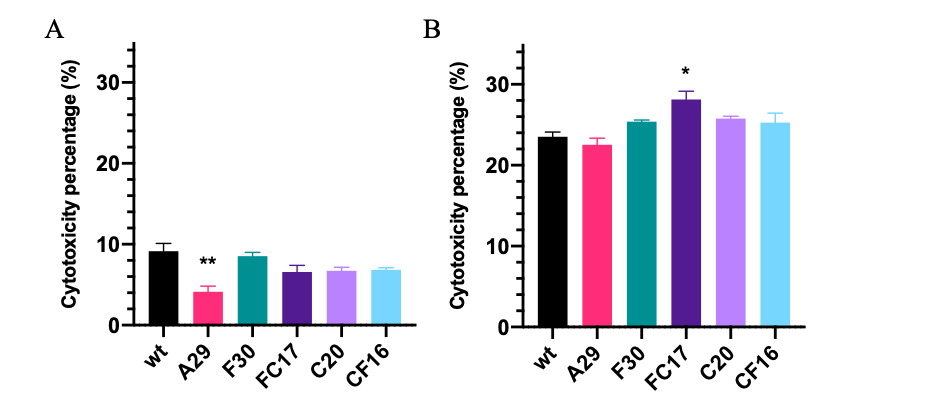

Supplement: FIG S5 [file mBio.03333-20-sf005.tif]
