# Supplementary material for: Genome-Wide Analysis of Experimentally Evolved Candida auris Reveals Multiple Novel Mechanisms of Multidrug Resistance
Source: mBio. 2021 Apr 5;12(2):e03333-20. doi: 10.1128/mBio.03333-20 (PMC8092288; doi:10.1128/mBio.03333-20)
Supplement: TABLE S1 [file mBio.03333-20-st001.docx]

**Table S1. All primers used in this study.** Primers are arranged per marker. ‘Purpose’ indicates whether the primer was used for PCR and sequencing (PCR/seq), CNV or expression analysis (qPCR) or allele-specific PCR (AS). Primer pairs for AS-PCR consist of a universal PCR-sequencing primer (indicated by ‘PCR/seq/AS’) or universal AS-primer (indicated by ‘AS’) and one allele-specific primer (indicated by ‘AS-wt’ for the wild type allele and ‘AS-mt’ for the mutant allele).

| **Marker** | **Purpose** | **Primer name** | **Sequence (5’-3’)** |
| --- | --- | --- | --- |
| *TAC1b* | PCR/seq/AS | CauTAC1b_B11220_PCR/Seq3_F | CGCTGCTCAAGTCAGGTAAGG |
|  | PCR/Seq | CauTAC1b_B11220_Seq3_R | AGGTGGCAAAGAAAGTCAACATG |
|  | AS-wt | CauTAC1b_SNP_F15F_R | CAATCCACTCAATACTTTGCGTATTG |
|  | AS-mt | CauTAC1b_SNP_F15/_R | TCAATCCACTCAATACTTTGCGTATTA |
|  | qPCR | CauQ-TAC1B-F1 | CACGCCCAATGGTTCGC |
|  | qPCR | CauQ-TAC1B-R1 | GGGTGAAGGTGCCTCCATG |
| *PEA2* | PCR/seq/AS | CauPEA2_B11220_PCR/Seq1_F | TAACACCTCGCCTAACTTGTGGT |
|  | PCR/seq | CauPEA2_B11220_Seq1_R | CTTTTCTCCCACATTGCATC |
|  | AS-wt | CauPEA2_SNP_D367D_R | GCAGAGTACCGTCTAGCATTAAATGA |
|  | AS-mt | CauPEA2_SNP_D367V_R | GCAGAGTACCGTCTAGCATTAAATGT |
| *MEC3* | PCR/seq/AS | CauMEC3_B11220_PCR/Seq1_F | CCAATGGGTATCATAAGTCAGCG |
|  | PCR/seq | CauMEC3_B11220_Seq1_R | GTATAACACCTCGACATCA |
|  | AS-wt | CauMEC3_SNP_A272A_R | GTGCTAACGATTTTCGGCG |
|  | AS-mt | CauMEC3_SNP_A272V_R | ACGTGCTAACGATTTTCGGCA |
| *FLO8* | AS | CauFLO8_SNP_Seq_R | GTGGACAGACACAGCTTGCTG |
|  | AS-wt | CauFLO8_SNP_Q384Q_F | GAACATGGGTATGCCTCGGC |
|  | AS-mt | CauFLO8_SNP_Q384*_F | ATGAACATGGGTATGCCTCGGT |
|  | PCR/seq | CauFLO8_B11220_Seq1_R | GTGGACAGACACAGCTTG |
|  | PCR/seq | CauFLO8_B11220_Seq1_F | AGTTCCCTCTTGATCAGA |
| *FKS1* | PCR/seq | CauFKS1_B11220_Seq2.2_F | ATTTCAGAAGGAACCTGG |
|  | PCR/seq | CauFKS1_B11220_Seq2.2_R | CGTTCCATTCGCTTATTC |
|  | AS | CauFKS1_B11220_Seq2_F | CTGCGAAATCAACACCTTTG |
|  | AS-wt | CauFKS1_SNP_M690M_R | CTTGTTCTTCTTGGATACTTACGTG |
|  | AS-mt | CauFKS1_SNP_M690I_R | GTTCTTGTTCTTCTTGGATACTTACGTA |
|  | AS-wt | CauFKS1_SNP_FL635FL_R | GTTGGCCGAATCTTACTTCCTC |
|  | AS-mt | CauFKS1_SNP_FL635L_R | GTTGGCCGAATCTTACTTCCTG |
| *ERG3* | PCR/seq | CauERG3_Seq2_F | TCAACGGATTCTCCAAGC |
|  | PCR/seq | CauERG3_B11220_Seq5_R | TGGAACCATCCGTCAACTG |
|  | AS^L207^ | CauERG3_PCR/Seq4_R | TACCATTGAATTTGGCTGC |
|  | AS-wt | CauERG3_SNP_L207L_F | CATCTACTTCATCCACCGCTAGC |
|  | AS-mt | CauERG3_SNP_L207I_F | GCATCTACTTCATCCACCGCTAGA |
|  | AS^W182^ | CauERG3_B11220_PCR/Seq1_F | CTCGTTTAGAGCTCGTTTTCAG |
|  | AS-wt | CauERG3_SNP_W182W’_R | GGAACTGTAACAATACGGCTCTC |
|  | AS-mt | CauERG3_SNP_W182*’_R | GGAACTGTAACAATACGGCTCTT |
| *ERG11* | PCR/seq | CauERG11_ Seq2_F | AACGAGAGAAGAAAGACCG |
|  | PCR/seq/AS | CauERG11_ B11220_PCR/Seq4_R | GCTGGTTTGGTGAAGAATTCGG |
|  | AS-wt | CauERG11_SNP_E429E_F | CCCACACAGATGGGGCG |
|  | AS-mt | CauERG11_SNP_E429*_F | GACCCACACAGATGGGGCT |
|  | qPCR | CauQ-ERG11-F | GTTTGCCTACGTGCAATTGG |
|  | qPCR | CauQ-ERG11-R | GTAGTCGACTGGTGGAAGCG |
| *CIS2* | PCR/seq | CauCIS2_B11220_Seq3_R | TTGTCTCGTTCTGCTTCCA |
|  | PCR/seq | CauCIS2_B11220_PCR/Seq3_F | TTTTTTCGCACCCATTTCG |
|  | AS | CauCIS2_B11220_Seq2_R | GCGGTGAGCTGAAAGAGAGC |
|  | AS-mt | CauCIS2_SNP_A27T_F | CGGCCATGGAGAACCA |
|  | AS-wt | CauCIS2_SNP_A27A_F | CGGCCATGGAGAACCG |
| *ACT1* | qPCR | CauQ-ACT1-F | GAAGGAGATCACTGCTTTAGCC |
|  | qPCR | CauQ-ACT1-R | GAGCCACCAATCCACACAG |
| *LSC2* | qPCR | CauQ-LSC2-F | TGTACCGACATGGAAGGAATTG |
|  | qPCR | CauQ-LSC2-R | TCACACCAAGACAGCTTTATCC |
| *UBC4* | qPCR | CauQ-UBC4-F | ACCTCAGCGGTTAACAAGAG |
|  | qPCR | CauQ-UBC4-R | CGAATCGGTGACGATCCATTA |
| *CDR1* | qPCR | CauQ-CDR1-F | GAAATCTTGCACTTCCAGCCC |
|  | qPCR | CauQ-CDR1-R | CATCAAGCAAGTAGCCACCG |
| *CDR2* | qPCR | CauQ-CDR2-F | GTCAACGGTAGCTGTGTG |
|  | qPCR | CauQ-CDR2-R | GTCCCTCCACCGAGTATGG |
